# Thigh Mass Case Report

**DOI:** 10.21980/J8QD3C

**Published:** 2022-10-15

**Authors:** Mary Rometti, Christopher Bryczkowski

**Affiliations:** * Rutgers Robert Wood Johnson Medical School, Department of Emergency Medicine, New Brunswick, NJ

## Abstract

Patients commonly present to the Emergency Department for the evaluation of soft tissues masses of various etiology. Point-of-care ultrasound (POCUS) can aid in the initial evaluation of these masses to begin narrowing a given differential. Soft tissue sarcomas are a malignant neoplasm that frequently present in an extremity, and require close follow-up for the evaluation of metastasis and possible resection, among other treatment options. Being able to effectively differentiate between infectious, inflammatory, benign, or potentially malignant pathology for undifferentiated soft tissue masses is critical for Emergency Medicine clinicians to ensure patients receive appropriate treatment and referrals for definitive care.

**Topics:**

Thigh mass, soft tissue mass, sarcoma, point-of-care ultrasound.

**Figure:**
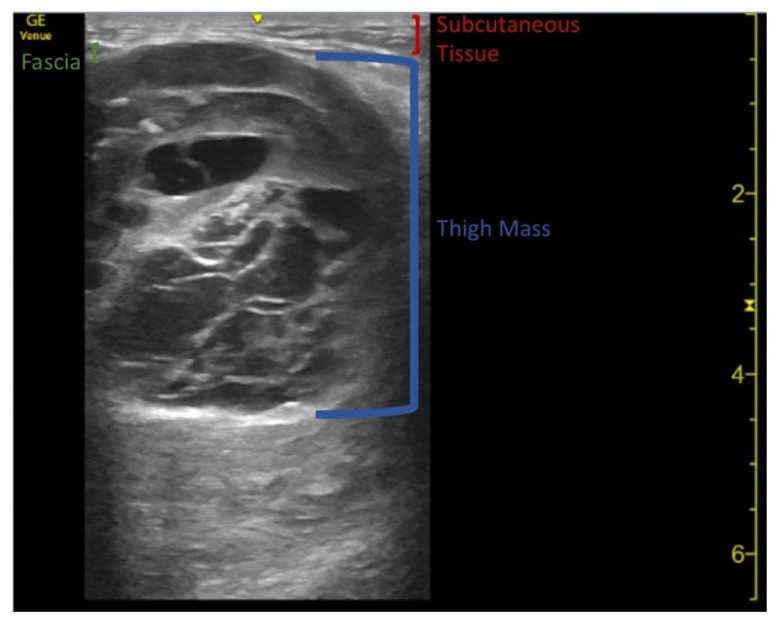
Video Link: https://youtu.be/IVAw2T8e5IY Doppler Video Link: https://youtu.be/XX8m1yv1hrA

**Figure:**
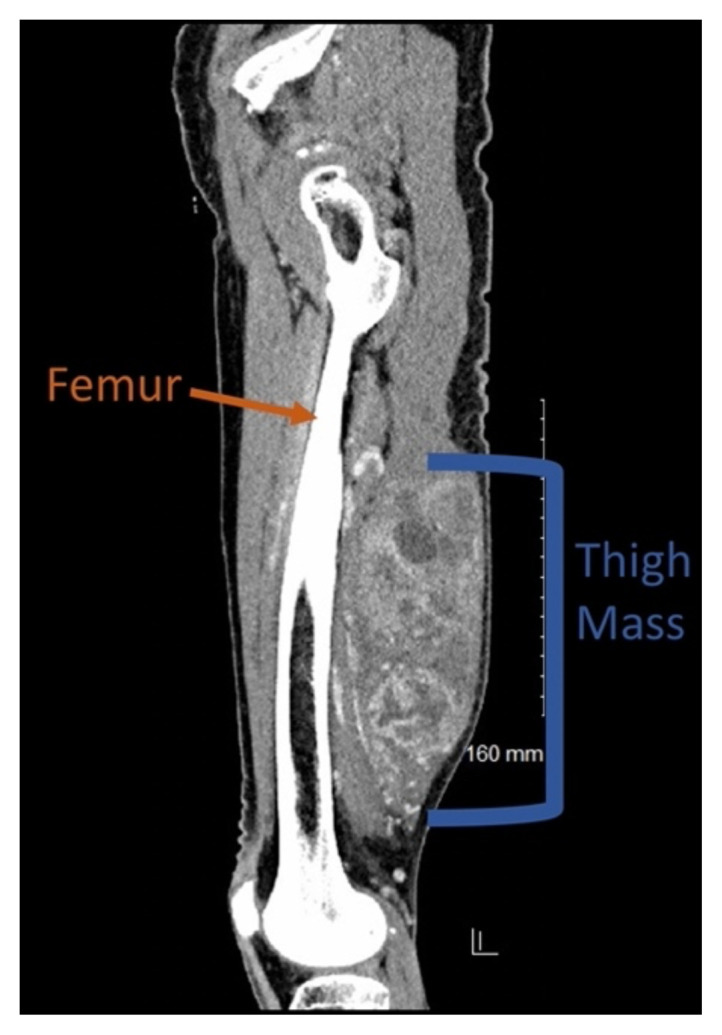


## Brief introduction

Soft tissue sarcomas are a type of mesenchymal tumor with over 70 different histological subtypes.^1^,^2^ This malignant neoplasm makes up about 1% of adult and 7–15% of pediatric malignancies.^1^,^3^–^5^ Most patients will be older, having a median age of 60 at the time of diagnosis.^1^ About 60% of soft tissue sarcomas are located in the extremities.^3^ Emergency medicine clinicians must be able to differentiate among soft tissue masses to ensure patients receive the appropriate treatment as well as follow-up.

## Presenting concerns and clinical findings

An 86-year-old male with no known medical problems presents to the Emergency Department (ED) with one week of pain and swelling of his right posterior upper leg. His family reports that he has had a small lump in the area for a long time, but the size seems to have increased. The patient describes pain in the affected area with ambulation as well as at rest. On physical exam, there is a firm, mildly tender, moderate-sized mass on the right posterior leg without fluctuance or erythema. Distal pulses are intact and his thigh compartments are soft.

## Significant findings

Point-of-care ultrasound (POCUS) demonstrates a large, subcutaneous mass with areas of mixed echogenicity. The mass contains fluid-filled, anechoic areas with internal septations and absent doppler flow. The majority of the mass appears isoechoic to the surrounding tissues with a hyperechoic border. Computed tomography (CT) of his right thigh shows a 16 x 8.1 x 9.5 cm heterogenous, complex mass within his hamstring muscles, inferior to the femur. His lab work was significant for a white blood cell (WBC) of 17.3 (10^3^/μL).

## Patient course

In the ED, surgical oncology was consulted. After bedside evaluation, the surgical team recommended that the patient should undergo an outpatient CT chest with contrast to look for metastatic spread and have clinic follow-up. About one month after initial presentation, the patient’s thigh mass was surgically removed. Pathology reports confirmed a high-grade pleomorphic sarcoma.

## Discussion

Evaluating a patient with a soft tissue mass in the ED is common. While the majority of these will be correctly diagnosed as inflammatory or infectious in etiology, it is important to keep a wide differential diagnosis. Malignant soft tissue masses may present with the appearance of a fluid collection, leading to the potential for a misdiagnosis.^6^,^7^ The most common misdiagnosis is a hematoma.^7^ Others include abscesses, ganglion cysts, foreign bodies, fat necrosis, and hernias.^7^ Because some of these alternate diagnoses involve bedside incision as part of routine care, correctly differentiating between infection, masses, and malignant pathology is vital for clinicians.^7^

Ultrasound is a useful modality to include in the initial workup of a patient with a soft tissue mass.^5^,^6^,^8^ Masses which display many of the following features require further studies to eliminate malignancy: tenderness, growth over time, size greater than 5 cm, location deep to the muscle fascia, heterogenous appearance, alteration of nearby anatomy, and those which demonstrate nonlinear vascular flow on Doppler.^6^,^7^

Originally, the mainstay of treatment for extremity soft tissue sarcomas was amputation.^3^ As treatment options have advanced, multimodality management has reduced the need for amputation.^3^ Of the various prognostic factors, including age over 50-years-old, histologic subtypes, and disease recurrence, the most significant factor is a microscopically positive margin.^3^ Positive margins are associated with an elevated risk leading to the potential for metastasis.^3^ The most common location for sarcomas to metastasize is the lung.^2^ If lung metastases are present, the medial survival is 15 months.^1^ Even with effective initial management, overall disease recurrence happens in about 50% of patients.^4^

This case demonstrates the importance of considering soft tissue sarcomas in the differential diagnosis of patients presenting with soft tissue masses. In addition, utilizing various imaging techniques including bedside ultrasound are helpful in narrowing down the diagnosis while examining for concerning neoplastic features. Additionally, providing appropriate subspecialty care and ensuring timely follow-up is also necessary. A limitation of this case is that the full treatment course and ultimate outcome for the patient is unknown. While a soft tissue sarcoma is not always an emergent diagnosis, failing to recognize a potentially malignant mass may have significant impacts on a patient’s life.

## Supplementary Information












