# Supplementary figures and images for: Thigh Mass Case Report
Source: J Educ Teach Emerg Med. 2022 Oct 15;7(4):V7–9. doi: 10.21980/J8QD3C (PMC10332667; doi:10.21980/J8QD3C)

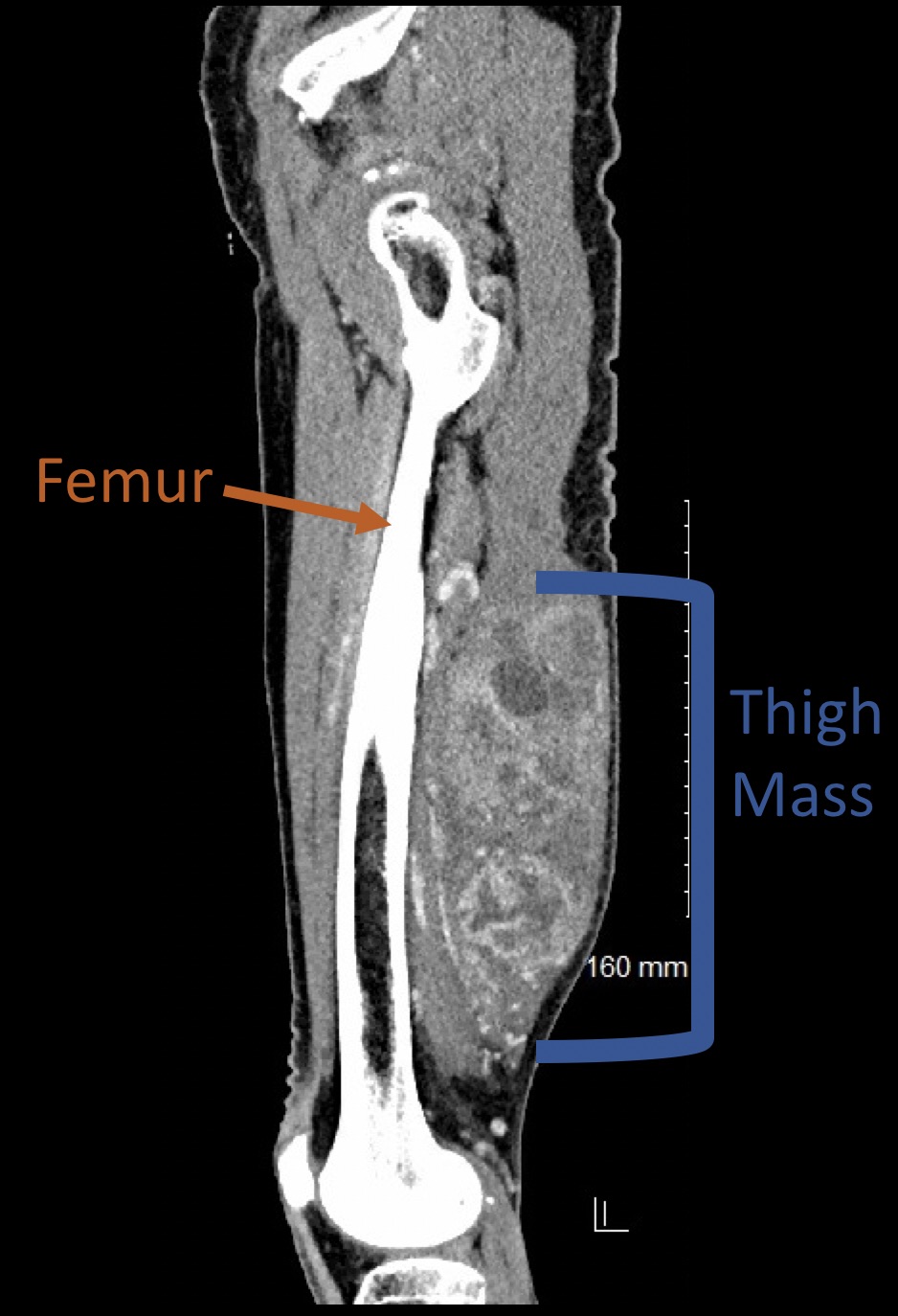

Supplement: Supplementary file 1 [file JETem-7-4-V7-supp1.jpg]

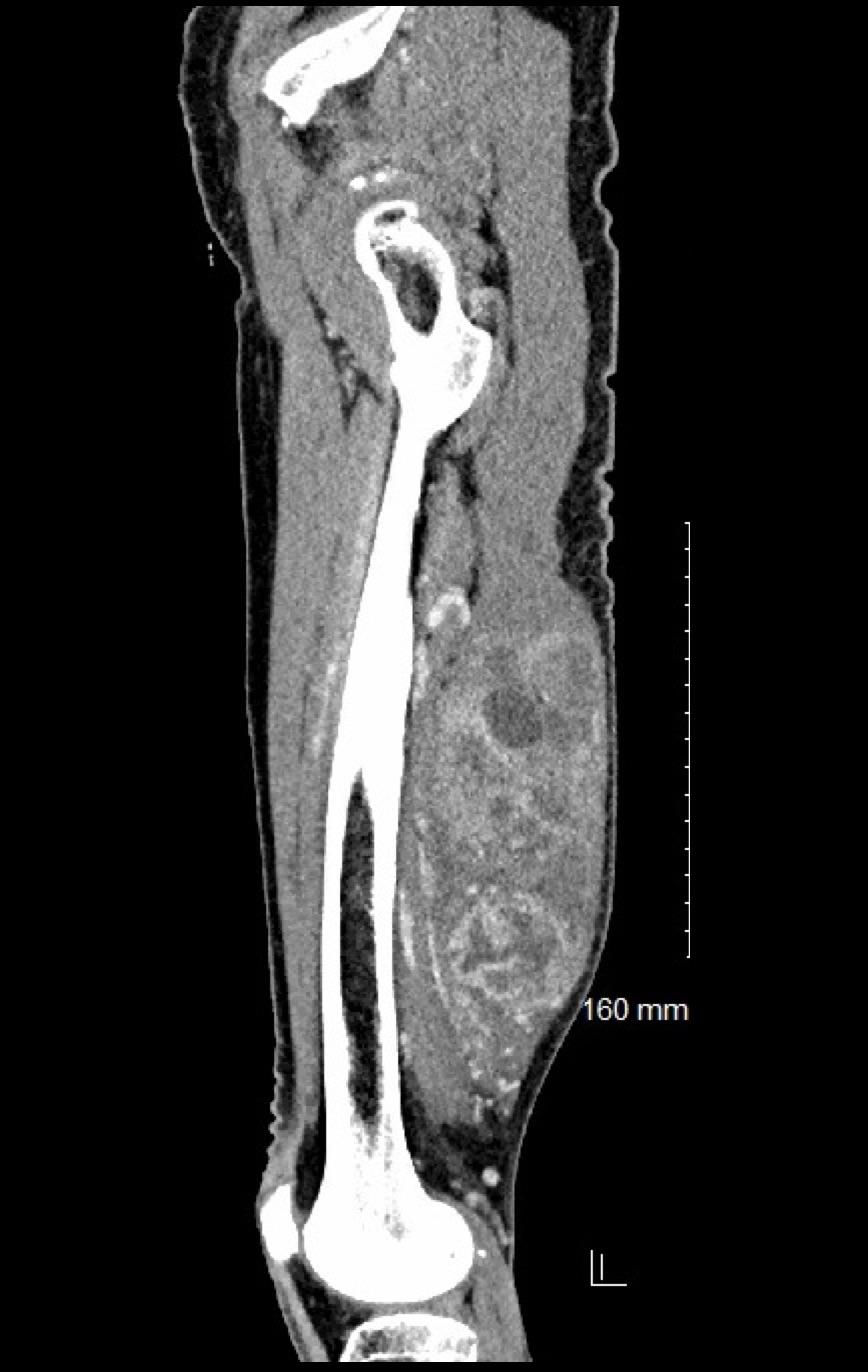

Supplement: Supplementary file 2 [file JETem-7-4-V7-supp2.jpg]

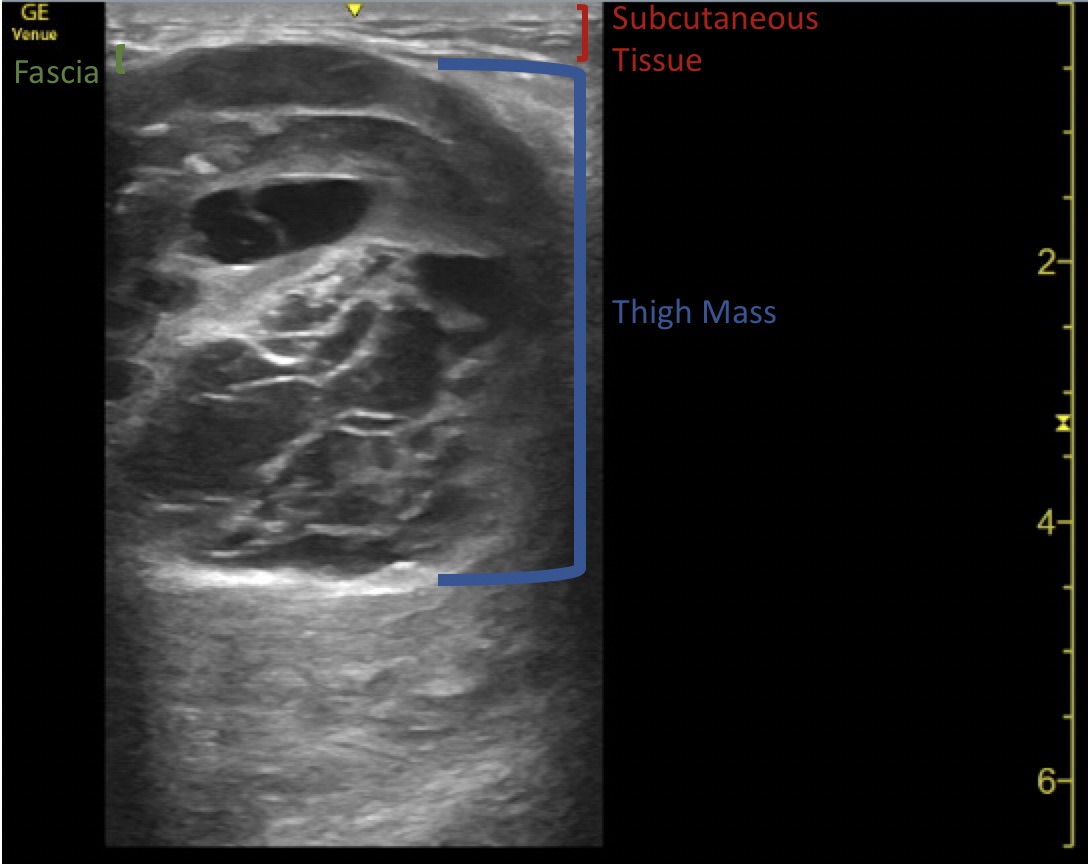

Supplement: Supplementary file 5 [file JETem-7-4-V7-supp5.jpg]

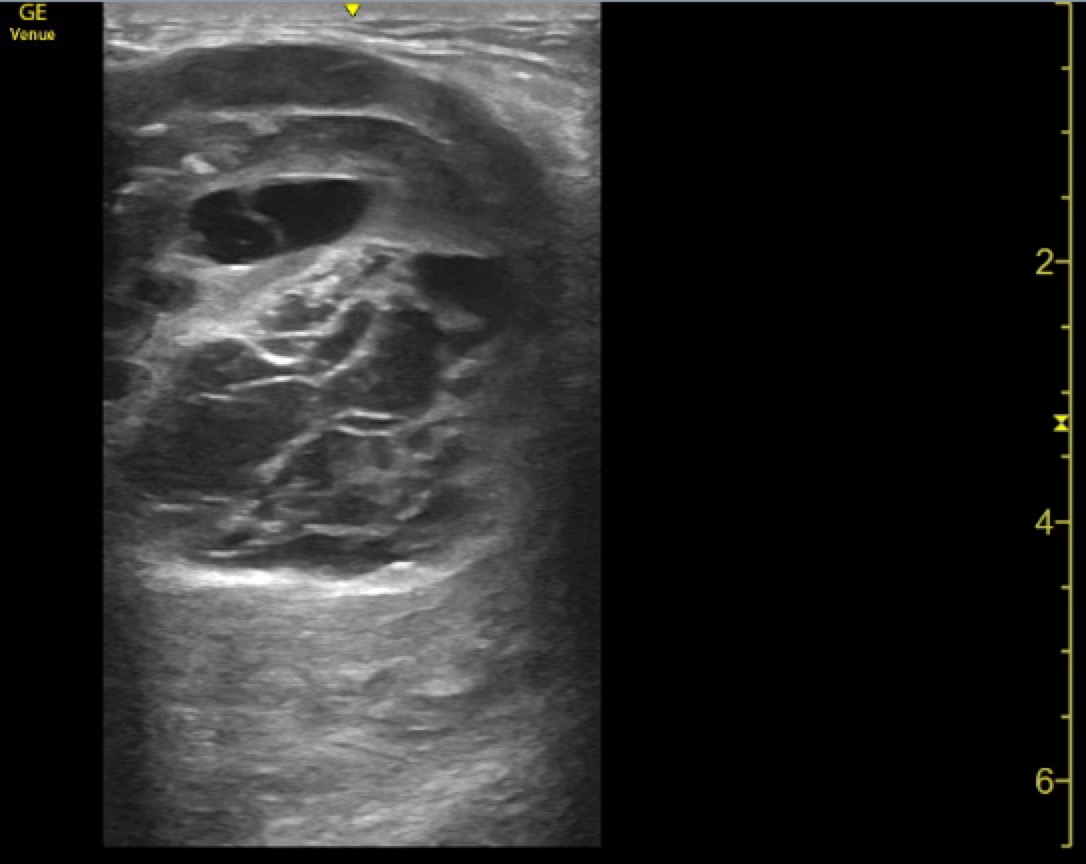

Supplement: Supplementary file 6 [file JETem-7-4-V7-supp6.jpg]
